# Analysis of the etiological characteristics of multidrug-resistant organisms and prognostic factors in ICU patients

**DOI:** 10.3389/fphys.2025.1658683

**Published:** 2025-09-26

**Authors:** Fengxia Du, Qun Ji, Ying Li, Jing Jia, Ruiping Xi

**Affiliations:** ^1^ Department of Hospital Infection Management, Inner Mongolia Baogang Hospital, Baotou, Inner Mongolia, China; ^2^ Department of Medical Insurance Office, Inner Mongolia Baogang Hospital, Baotou, Inner Mongolia, China; ^3^ Department of General Practice, Inner Mongolia Baogang Hospital, Baotou, Inner Mongolia, China; ^4^ Department of Anesthesiology and Surgery, Inner Mongolia Baogang Hospital, Baotou, Inner Mongolia, China; ^5^ Clinical Laboratory, The 969th Hospital of PLA, Hohhot, Inner Mongolia, China

**Keywords:** multidrug-resistant organism, intensive care unit, pathogenic characteristics, poor prognosis, influencing factors

## Abstract

**Background:**

Multidrug-resistant organism (MDRO) infections contribute to high mortality in intensive care unit (ICU) patients, yet their specific pathogen profile and mortality risk factors are inadequately characterized.

**Objective:**

In this study, we aim to investigate MDRO infections in ICU patients, identify prevalent pathogens, and evaluate risk factors associated with 28-day mortality.

**Methods:**

A retrospective study of 260 ICU patients with MDRO infections (resistant to ≥3 antimicrobial classes) analyzed the specimen types, infection sites, and pathogens. Patients were grouped (survival group and non-survival group) based on 28-day survival outcomes. Multivariate logistic regression identified prognostic factors, and the model’s validity, fit, and discriminatory power were assessed.

**Results:**

In ICU patients with MDRO infections, sputum was the most common test specimen, with the respiratory system as the main infection site. Pathogenic bacteria primarily included *Escherichia coli*, *Klebsiella pneumoniae*, *Pseudomonas aeruginosa*, and *Staphylococcus aureus*. Sixty-seven patients died within 28 days after enrollment (mortality rate: 25.77%). ICU length of stay [odds ratio (OR): 1.141; 95% confidence interval (CI): 1.020–1.275], Acute Physiology and Chronic Health Evaluation II (APACHE II) score upon admission (OR: 1.496; 95% CI: 1.261–1.775), comorbidity with cardiovascular and cerebrovascular diseases (OR: 4.620; 95% CI: 1.665–12.821), comorbidity with pulmonary diseases (OR: 4.150; 95% CI: 1.722–10.000), duration of mechanical ventilation >7 days (OR: 3.457; 95% CI: 1.502–7.955), and number of invasive procedures (OR: 1.845; 95% CI: 1.239–2.748) were independent risk factors for poor prognosis in ICU patients with MDRO infections. A logistic regression equation was developed: logistic regression equation = −20.646 + 0.132X1 (ICU stay) + 0.403X2 (APACHE II score) + 1.530X3 (cardiovascular/cerebrovascular comorbidities) + 1.423X4 (pulmonary comorbidities) + 1.240X5 (mechanical ventilation >7 days) + 0.613X6 (invasive procedures). The model was statistically significant (likelihood ratio chi-square test, *P* < 0.05) and demonstrated a good fit (Hosmer–Lemeshow test, *P* > 0.05). The area under the curve (AUC) was 0.913 (0.85 ≤ AUC <0.95), with 65.67% sensitivity, 93.78% specificity, and 86.54% accuracy in predicting the survival/death risk in MDRO-infected patients, indicating strong discriminatory power.

**Conclusion:**

ICU patients with MDRO infections exhibit diverse pathogens. Prompt preventive and control measures should be implemented based on the characteristics of MDRO infections and high-risk factors associated with prognosis to reduce the risk of death following MDRO infections.

## Introduction

Healthcare and public health organizations worldwide prioritize preventing and controlling the spread of infections, especially considering that infections involving multidrug-resistant organisms (MDROs) are linked to increased mortality and healthcare costs (AlRawashdeh et al., 2024). International subject matter experts, through a joint initiative by the European Centre for Disease Prevention and Control and the U.S. Centers for Disease Control and Prevention, have created a standardized international definition for acquired resistance characteristics. MDROs are defined as strains that exhibit resistance to three or more different classes of antibiotics simultaneously (Abdeta et al., 2021). These resistant organisms are responsible for a significant proportion of healthcare-associated infections in intensive care unit (ICU) patients (Wang et al., 2023). It is important to note that colonization refers to the presence of bacteria on body surfaces (such as the skin and respiratory tract) without causing disease, whereas infection involves the invasion and proliferation of pathogens within the body, leading to tissue damage and clinical symptoms (Almohaya et al., 2024; Liu et al., 2024). Patients admitted to the ICU are typically in severe condition and necessitate multiple invasive interventions. Consequently, the ICU is regarded the unit most affected by MDRO infections in hospital settings (Golli et al., 2022).

Organisms resistant to carbapenem antibiotics and those producing carbapenemases, such as *Escherichia coli* (*E. coli*), *Klebsiella pneumoniae* (*K. pneumoniae*), *Staphylococcus aureus* (*S. aureus*), and *Pseudomonas aeruginosa* (*P. aeruginosa*), have become one of the most important causes of hospital- and community-acquired infections. They can cause urinary tract, respiratory tract, bloodstream, abdominal, and wound infections, along with meningitis and malignant otitis externa (Abdeta et al., 2021). *E. coli* and *K. pneumoniae* are common pathogens in urinary tract, bloodstream, and respiratory infections, often showing high resistance to carbapenems, which are considered last-line antibiotics (Siopi et al., 2024; Cabrera et al., 2025). *P. aeruginosa* is notorious for its inherent and acquired resistance to multiple antibiotics (Siopi et al., 2024), complicating treatment for critically ill patients. *S. aureus*, particularly methicillin-resistant *S. aureus* (MRSA), is a common cause of hospital-acquired infections associated with significant morbidity and mortality (Galfo et al., 2025). Early prediction of the MDRO infection risk in patients enables timely interventions, effectively reducing colonization rates among ICU patients. This strategy also minimizes the risks of both endogenous infections and cross-transmission between patients and healthcare workers (Salomao et al., 2020). Nonetheless, there is a delay in ascertaining the infection status of patients because the outcomes of drug sensitivity testing and microbial culturing take 24–72 h to become available (Wang et al., 2023). Considering the promising advantages associated with predictive models in the context of MDRO, numerous research workers formulated diverse models utilizing logistic regression to forecast the likelihood of MDRO infection (Gonzalez Del Castillo et al., 2020; Wang et al., 2020).

Given this backdrop, despite the recognized threat of MDROs in ICUs, knowledge gaps remain regarding the precise etiological characteristics and prognostic factors of these infections. Previous studies have provided valuable insights, but regional differences, changing resistance patterns, and the dynamic nature of ICU populations necessitate ongoing research. In this study, we aim to fill these gaps by evaluating pathogen distribution, resistance patterns, and prognostic factors in ICU patients with MDRO infections. In particular, we aim to assess the prevalence of key bacterial species, their antimicrobial resistance profiles, and factors influencing 28-day mortality. By achieving these objectives, we hope to contribute to the development of more effective infection control strategies and to the optimization of antimicrobial stewardship in critical care settings.

## Materials and methods

### Ethics statement

Ethical approval for the study was granted by the Ethics Committee of The 969th Hospital of PLA, and informed consent was obtained from all participants.

### Study site and date

The study was conducted at The 969th Hospital of PLA, a tertiary comprehensive hospital located in Harbin, China. The hospital has approximately 450 inpatient beds and is equipped with several ICUs, including general ICU and CCU units, providing adequate bed capacity for critically ill patient care. Medical records of hospitalized patients with MDRO infections in the ICU were collected. Data were collected from January 2020 to December 2022.

### Study participants

A retrospective research method was used in this study to identify 260 subsequent study subjects based on inclusion and exclusion criteria. The cohort comprised 180 male and 80 female subjects, aged 46–81 years, with a mean age of 61.64 ± 6.60 years.

### Inclusion and exclusion criteria

Inclusion criteria were as follows: 1. patients who met the diagnostic criteria for hospital-acquired infections (Ministry of Health of the People’s Republic of China, 2001) and had a hospital stay of no less than 48 h, 2. those whose etiological test results from submitted samples indicated MDRO, and 3. those aged >18 years. Exclusion criteria were as follows: 1. patients with ICU length of stay <48 h, 2. those with HAIs or MDRO infections prior to ICU admission, 3. those with infection cases where collected samples were suspected of contamination, 4. those with resistant bacterial strain cultures determined to be colonization by infectious disease experts and ICU physicians together, 5. those with inflammatory responses caused by other non-bacterial biological factors, and 6. those with incomplete clinical data.

### Etiological testing

ICU healthcare providers strictly adhered to laboratory standard operating procedures for the collection and transportation of etiological specimens from different infection sites. Specimens included sputum, blood, urine, ascites, drainage fluid, pus, alveolar lavage fluid, and secretions. The bacterial identification instrument used in this study was the internationally recognized bioMérieux VITEK2-COMPACT (bioMérieux, France). Bacterial identification and susceptibility cards used original French bioMérieux reagents, with GN13 for Gram-negative bacteria and GP67 for Gram-positive bacteria. Susceptibility testing included the following tests: (1) the disk diffusion method (K-B method); (2) susceptibility testing using susceptibility identification cards compatible with the VITEK2-COMPACT automated microbial identification system. AST-GN13 was used for Gram-negative bacteria, and AST-GP67 or AST-GP68 for Gram-positive bacteria; (3) Extended-Spectrum Beta-Lactamase detection test: identification of ESBL-producing strains was performed using an instrumental method based on the principle of determining the minimum inhibitory concentration (MIC) of either ceftazidime (30 μg/disk) or cefotaxime (30 μg/disk), with a reduction in MIC by more than three serial dilutions after the addition of clavulanic acid, indicating the production of ESBLs by the bacteria; (4) MRSA test: cefoxitin (30 μg/disk) was used, and any inhibition zone diameter ≤21 mm was considered MRSA; (5) carbapenem resistance: resistance to any carbapenem drug, such as imipenem, meropenem, or ertapenem, was considered carbapenem resistance. Relevant testing procedures and interpretation of susceptibility test results followed the 2015 Clinical and Laboratory Standards Institute guidelines (CLSI M100-S25) (CLSI M100-S25, 2015), along with the collection, processing, culture, and identification requirements specified in the “National Clinical Laboratory Procedures” (Clinical Laboratory Center and Ministry of Health of the People’s Republic of China, 2016) and the “Standards for the Performance of Antimicrobial Susceptibility Testing” (National Health and Family P lanning Commission of the People’s Republic of China, 2017) to ensure consistency in specimen collection methods and homogeneity in specimen identification. Definitions of related MDROs are as follows: MDROs are bacteria that demonstrate resistance to three or more antimicrobial drugs commonly used in clinical practice. Common MDROs include carbapenem-resistant *E. coli* (CREO), carbapenem-resistant *K. pneumoniae* (CRKP), carbapenem-resistant *P. aeruginosa* (CRPA), MRSA, and carbapenem-resistant *Acinetobacter baumannii* (*A. baumannii*) (CRAB).

### Data collection

We analyzed the types and distribution of specimens collected for testing, the distribution of infection sites, and the types and distribution of pathogenic bacteria among ICU patients with MDRO infections. A retrospective study approach was applied to collect clinical data on ICU patients with MDRO infections using the hospital’s electronic medical record system, hospital infection management monitoring system, and ICU monitoring system. Data collected included baseline characteristics (gender, age, and ICU length of stay), severity scores [Acute Physiology and Chronic Health Evaluation II (APACHE II) score upon admission], whether surgery was performed, comorbidities (cardiovascular and cerebrovascular diseases, diabetes, hypertension, cancer, pulmonary disease, or liver diseases), enteral nutrition, use of corticosteroid medications, invasive procedures (nasogastric tube placement, urinary catheter placement, tracheotomy, mechanical ventilation, and central venous catheterization), and 28-day prognosis of patients (Feng et al., 2012) (death within 28 days after enrollment, including in-hospital death and out-of-hospital follow-up death). Patients were classified into survival and non-survival groups based on 28-day mortality.

### Statistical methods

Data analysis was performed using SPSS 21.0 software (IBM Corp, Armonk, N.Y, United States of America). Measurement data (age, ICU length of stay, admission APACHE II score, and number of invasive procedures) were depicted as mean ± standard deviation ( $\bar{x}$ ± s) and compared with an independent samples *t*-test. Categorical data were reported as percentages (%) and compared with the *χ* ^*2*^ test. Multivariate logistic regression analysis was performed to identify factors influencing the prognosis of ICU patients with MDRO infections by evaluating the odds ratios (ORs) and 95% confidence intervals (95% CIs) using SPSS 21.0 software. A logistic regression model was established, and its overall validity was tested using the likelihood ratio chi-square test. The goodness-of-fit was tested using the Hosmer–Lemeshow test, and the model’s discriminatory ability was assessed using the receiver operating characteristic (ROC) curve. *P* < 0.05 was considered statistically significant.

## Results

### Specimen type and distribution

A total of 260 multidrug-resistant strains were detected, primarily from sputum cultures, accounting for 145 strains (55.77%), followed by 32 strains from blood (12.31%), 27 strains from midstream urine (10.38%), 19 strains from ascites (7.31%), 17 strains from drainage fluid (6.54%), 7 strains from secretions (2.69%), 5 strains from pus (1.92%), 5 other specimens (1.92%), and 3 strains from alveolar lavage fluid (1.15%) (Table 1).

**TABLE 1:** Specimen type and distribution collected for testing.

Specimen type	Number collected for testing	Percentage
Sputum	145	55.77
Blood	32	12.31
Mid-segment urine	27	10.38
Abdominal fluid	19	7.31
Drainage fluid	17	6.54
Secretion	7	2.69
Pus	5	1.92
Alveolar lavage fluid	3	1.15
Others	5	1.92

### Infection site distribution

From the perspective of the infection sites of pathogenic bacteria, the most common site of infection was the respiratory system (151 strains, 58.08%), followed by the hematological system (35 strains, 13.46%) (Table 2).

**TABLE 2:** Infection site distribution.

Site of infection	Number	Percentage
Respiratory system	151	58.08
Blood system	35	13.46
Urinary system	22	8.46
Digestive system	22	8.46
Surgical incision	21	8.08
Skin and soft tissue systems	9	3.46

### Pathogenic bacterium profiles and their prevalence patterns

A total of 260 multidrug-resistant strains were isolated, and Gram-negative bacteria accounted for 171 infections (65.77%). The three most prevalent species were as follows: *E. coli* (68 strains, accounting for 26.15%), *K. pneumoniae* (39 strains, accounting for 15.00%), and *P. aeruginosa* (23 strains, accounting for 8.85%). Meanwhile, Gram-positive bacteria contributed to 89 strains (34.23%), with *S. aureus* (49 strains, accounting for 18.85%), *Streptococcus pneumoniae* (*S. pneumoniae*) (12 strains, accounting for 4.62%), and *Staphylococcus haemolyticus* (10 strains, accounting for 3.85%) as the dominant pathogens (Table 3).

**TABLE 3:** Pathogenic bacteria and their prevalence patterns.

Pathogenic bacteria		Number	Percentage
Gram-negative bacteria	Total Gram-negative	170	35.77
	*Escherichia coli*	68	26.15
	*Klebsiella pneumoniae*	39	15.00
	*Pseudomonas aeruginosa*	23	8.85
	*Acinetobacter baumannii*	14	5.38
	*Haemophilus influenzae*	13	5.00
	*Klebsiella aerogenes*	2	0.77
	*Klebsiella oxytoca*	2	0.77
	Other negative bacteria	10	3.85
Gram-positive bacteria	Total Gram-positive	89	34.23
	*Staphylococcus aureus*	49	18.85
	*Streptococcus pneumoniae*	12	4.62
	*Staphylococcus haemolyticus*	10	3.85
	*Staphylococcus hominis*	5	1.92
	*Staphylococcus epidermidis*	4	1.54
	*Enterococcus faecium*	3	1.15
	*Staphylococcus kongii*	2	0.77
	Other positive bacteria	4	1.54

### Drug resistance of major pathogens

The drug resistance testing results of the four most prevalent pathogens were analyzed. Susceptibility test results showed that *E. coli* strains had low resistance rates to imipenem, amikacin, piperacillin/tazobactam, and cefoxitin, with a resistance rate to ampicillin exceeding 80%. *K. pneumoniae* had low resistance rates to amikacin, imipenem, and cefoxitin, with resistance rates to ampicillin/sulbactam, trimethoprim–sulfamethoxazole, and cefazolin exceeding or approaching 40%. *P. aeruginosa* isolates showed low resistance only to amikacin, with resistance rates to cefoxitin and cefazolin approaching or reaching 100%. *S. aureus* isolates showed high sensitivity to linezolid (resistance rate: 0%) and the highest resistance rate to penicillin (95.92%). Detailed results are shown in Table 4.

**TABLE 4:** Drug-resistance profiles of *Escherichia coli*, *Klebsiella pneumoniae*, *Pseudomonas aeruginosa*, and *Staphylococcus aureus*.

*Escherichia coli* (n = 68)		*Klebsiella pneumoniae* (n = 39)		*Pseudomonas aeruginosa* (n = 23)		*Staphylococcus aureus* (n = 49)	
Antimicrobial drug	Number of resistant strains (resistance rate %)	Antimicrobial drug	Number of resistant strains (resistance rate %)	Antimicrobial drug	Number of resistant strains (resistance rate %)	Antimicrobial drug	Number of resistant strains (resistance rate %)
Imipenem	1 (1.47)	Amikacin	2 (5.13)	Amikacin	1 (4.35)	Linezolid	0 (0.00)
Amikacin	3 (4.41)	Imipenem	2 (5.13)	Tobramycin	1 (4.35)	Vancomycin	1 (2.04)
Piperacillin/tazobactam	3 (4.41)	Cefotetan	2 (5.13)	Gentamicin	2 (8.70)	Rifampicin	2 (4.08)
Cefotetan	4 (5.88)	Piperacillin/tazobactam	3 (7.69)	Levofloxacin	2 (8.70)	Moxifloxacin	4 (8.16)
Tobramycin	11 (16.18)	Tobramycin	6 (15.39)	Piperacillin/tazobactam	2 (8.70)	Levofloxacin	5 (10.20)
Cefepime	13 (19.12)	Cefepime	7 (17.95)	Cefepime	2 (8.70)	Ciprofloxacin	8 (16.33)
Ceftazidime	17 (25.00)	Levofloxacin	8 (20.51)	Ceftazidime	3 (13.04)	Gentamicin	9 (18.37)
Aztreonam	22 (32.35)	Gentamicin	9 (23.08)	Ciprofloxacin	3 (13.04)	Tetracycline	9 (18.37)
Gentamicin	33 (48.53)	Ceftazidime	10 (25.64)	Imipenem	4 (17.39)	Oxacillin	13 (26.53)
Levofloxacin	35 (51.47)	Ciprofloxacin	11 (28.20)	Cefotetan	22 (95.65)	Compound sulfamethoxazole	13 (26.53)
Ceftriaxone	36 (52.94)	Aztreonam	12 (30.77)	Cefazolin	23 (100.00)	Clindamycin	20 (40.81)
Ciprofloxacin	37 (54.41)	Ceftriaxone	14 (35.90)			Erythromycin	37 (75.51)
Cefazolin	40 (58.82)	Compound sulfamethoxazole	15 (38.46)			Penicillin	47 (95.92)
Ampicillin/sulbactam	41 (60.29)	Ampicillin/sulbactam	16 (41.02)				
Compound sulfamethoxazole	43 (63.23)	Cefazolin	15 (38.46)				
Ampicillin	59 (86.76)						

### Prognostic factor analysis

The cohort of 260 MDRO-infected ICU patients comprised 193 survivors (74.23%) in the survival group and 67 non-survivors (25.77%) in the non-survival group. Univariate comparative analysis revealed significant disparities (*P* < 0.05) between these groups across multiple clinical parameters, including ICU length of stay, APACHE II scores upon admission, concurrent cardiovascular/cerebrovascular pathologies, comorbidity with diabetes, comorbidity with pulmonary diseases, enteral nutrition, use of corticosteroid medications, duration of mechanical ventilation, central venous catheterization, and the number of invasive procedures (invasive procedures included mechanical ventilation, indwelling urinary catheter, indwelling central venous catheter, indwelling gastric tube, and indwelling drainage tube). No significant intergroup differences were found in gender, age, surgical procedures performed, comorbidity with hypertension, comorbidity with cancer, comorbidity with liver diseases, nasogastric tube placement, urinary catheter placement, tracheotomy, and classification of pathogenic bacteria (*P* > 0.05) (Table 5).

**TABLE 5:** Univariate analysis of factors influencing the prognosis of MDRO infection in ICU patients.

Indicator		Survival group (n = 193)	Non-survival group (n = 67)	*P*
Gender, n (%)	Male	135 (69.95)	45 (67.16)	0.671
	Female	58 (30.05)	22 (32.84)	
Age (year)		61.48 ± 6.42	62.09 ± 7.10	0.517
ICU length of stay (d)		14.48 ± 2.96	16.31 ± 4.16	<0.001
APACHE Ⅱ score upon admission (point)		16.77 ± 2.31	19.30 ± 2.19	<0.001
Surgery, n (%)	Yes	140 (72.54)	46 (68.66)	0.544
	No	53 (27.46)	21 (31.34)	
Comorbidity with cardiovascular and cerebrovascular diseases, n (%)	Yes	119 (61.66)	59 (88.06)	<0.001
	No	74 (38.34)	8 (11.94)	
Comorbidity with diabetes, n (%)	Yes	42 (21.76)	23 (34.33)	0.041
	No	151 (78.24)	44 (65.67)	
Comorbidity with hypertension, n (%)	Yes	101 (52.33)	42 (62.69)	0.142
	No	92 (47.67)	25 (37.31)	
Comorbidity with cancer, n (%)	Yes	23 (11.92)	12 (17.91)	0.216
	No	170 (88.08)	55 (82.09)	
Comorbidity with pulmonary diseases, n (%)	Yes	93 (48.19)	57 (85.07)	<0.001
	No	100 (51.81)	10 (14.93)	
Comorbidity with liver diseases, n (%)	Yes	27 (13.99)	13 (19.40)	0.290
	No	166 (86.01)	54 (80.60)	
Enteral nutrition, n (%)	Yes	170 (88.08)	65 (97.01)	0.033
	No	23 (11.92)	2 (2.99)	
Use of corticosteroid medications, n (%)	Yes	175 (90.67)	66 (98.51)	0.034
	No	18 (9.33)	1 (1.49)	
Nasogastric tube placement, n (%)	Yes	176 (91.19)	64 (95.52)	0.252
	No	17 (8.81)	3 (4.48)	
Urinary catheter placement, n (%)	Yes	190 (98.45)	67 (100.00)	0.305
	No	3 (1.55)	0 (0.00)	
Tracheotomy, n (%)	Yes	99 (51.30)	42 (62.69)	0.107
	No	94 (48.70)	25 (37.31)	
Duration of mechanical ventilation (d) , n (%)	≤7	106 (54.92)	16 (23.88)	<0.001
	>7	87 (45.08)	51 (76.12)	
Central venous catheterization, n (%)	Yes	176 (91.19)	66 (98.51)	0.042
	No	17 (8.81)	1 (1.49)	
Number of invasive procedures (times)		3.21 ± 0.64	3.80 ± 1.39	<0.001
Classification of pathogenic bacteria, n (%)				0.563
Gram-negative bacteria		125 (64.77)	46 (68.66)	
Gram-positive bacteria		68 (35.23)	21 (31.34)	

### Multivariate logistic regression of prognostic factors

Whether MDRO-infected patients survived after 28 days was used as the dependent variable (0 = survived; 1 = did not survive). Factors with statistically significant differences in the univariate analysis results in Table 5 were used as independent variables (ICU length of stay: continuous variable; admission APACHE II score: continuous variable; comorbid cardiovascular and cerebrovascular diseases: no = 0 and yes = 1; comorbid diabetes: no = 0 and yes = 1; comorbid pulmonary diseases: no = 0 and yes = 1; enteral nutrition: no = 0 and yes = 1; use of corticosteroids: no = 0 and yes = 1; duration of mechanical ventilation: >7 days = 1 and ≤7 days = 0; central venous catheterization: no = 0 and yes = 1; number of invasive procedures: continuous variable) and included in a binary logistic regression analysis model. The enter method was used to screen relevant risk factors, with a significance level of 0.05 for introducing variables. The logistic regression model identified six independent predictors of poor prognosis (*P* < 0.05): ICU length of stay (OR: 1.141; 95% CI: 1.020–1.275), APACHE II score upon admission (OR: 1.496; 95% CI: 1.261–1.775), comorbidity with cardiovascular and cerebrovascular diseases (OR: 4.620; 95% CI: 1.665–12.821), comorbidity with pulmonary diseases (OR: 4.150; 95% CI: 1.722–10.000), duration of mechanical ventilation >7 days (OR: 3.457; 95% CI: 1.502–7.955), and number of invasive procedures (OR: 1.845; 95% CI: 1.239–2.748) (Table 6).

**TABLE 6:** Multivariate analysis of factors influencing the prognosis of MDRO infection in ICU patients.

Factor	B	Standard error	Wald	*P*	Odds ratio	95% confidence interval
Length of ICU stay	0.132	0.057	5.359	0.021	1.141	1.02–1.275
APACHE II score on admission	0.403	0.087	21.300	<0.001	1.496	1.261–1.775
Comorbid cardiovascular and cerebrovascular diseases	1.530	0.521	8.636	0.003	4.620	1.665–12.821
Comorbid diabetes	0.855	0.451	3.590	0.058	2.351	0.971–5.692
Comorbid pulmonary diseases	1.423	0.449	10.054	0.002	4.150	1.722–10.000
Enteral nutrition	1.389	0.925	2.258	0.133	4.013	0.655–24.577
Use of corticosteroids	2.446	1.337	3.346	0.067	11.546	0.84–158.746
Mechanical ventilation duration >7 days	1.240	0.425	8.509	0.004	3.457	1.502–7.955
Central venous catheterization	1.509	1.162	1.687	0.194	4.524	0.464–44.101
Number of invasive procedures	0.613	0.203	9.098	0.003	1.845	1.239–2.748
Constant	−20.646	3.115	43.919	0.000	0.000	

### Model establishment

Binary logistic regression analysis screened out ICU length of stay, APACHE II score, comorbid cardiovascular and cerebrovascular diseases, comorbid pulmonary diseases, duration of mechanical ventilation >7 days, and number of invasive procedures as factors with statistically significant differences (P < 0.05). The following logistic regression equation was derived: logistic regression equation (P) = −20.646 + 0.132X1 + 0.403X2 + 1.530X3 + 1.423X4 + 1.240X5 + 0.613X6, where X1 refers to the ICU length of stay, X2 refers to the APACHE II score, X3 refers to the comorbid cardiovascular and cerebrovascular diseases, X4 refers to the comorbid pulmonary diseases, X5 refers to the duration of mechanical ventilation >7 days, and X6 refers to the number of invasive procedures. The overall model validity was tested using the likelihood ratio chi-square test, with a chi-square value of 127.465 and a *P* value <0.001, indicating statistical significance in model establishment. The Hosmer–Lemeshow test showed a chi-square value of 3.606 and a *P* value of 0.891 (*P* > 0.05), indicating sufficient data information extraction and good model fit.

**FIGURE 1:**
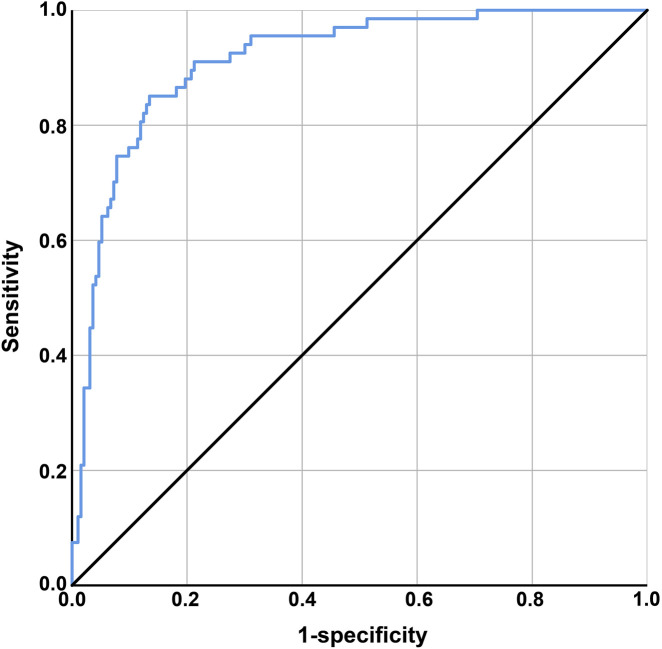
ROC curve.

Discriminatory ability was assessed using the ROC curve. The area under the curve (AUC) was defined as 1, with a larger AUC indicating higher clinical accuracy and better discriminatory ability. The X-axis was set as 1–specificity, where higher accuracy is indicated as the X-axis approaches zero, and the Y-axis was set as sensitivity, with higher accuracy represented by a larger Y-axis value. The AUC of the ROC curve was 0.913 (0.85 ≤ AUC <0.95), with a standard error of 0.019, a 95% confidence interval of 0.875–0.951, and a significance level of *P* < 0.001, indicating good discriminatory ability of the model, as shown in Figure 1. The model’s predictions and actual occurrences are shown in Table 7. The Youden index was used to measure the optimal cutoff value, with a maximum Youden index of 0.595, sensitivity of 65.67%, specificity of 93.78%, positive predictive value of 78.57%, negative predictive value of 88.73%, and an overall predictive accuracy of 86.54% for the risk probability of survival and death in patients with MDRO infections, indicating good model predictive accuracy and certain clinical guiding significance.

**TABLE 7:** Model predictions versus actual outcomes.

Prediction observation		Predicted prognosis		
		Survival (negative)	Death (positive)	Total
Observed prognosis	Survival (negative)	181	12	193
	Death (positive)	23	44	67
	Total	204	56	260

## Discussion

In this study, we evaluated MDRO infections in ICU patients, identified prevalent pathogens, and assessed risk factors associated with 28-day mortality. The results indicated that the respiratory system was the primary site of infection among MDRO infections in the ICU. Additionally, among Gram-negative bacteria, *E. coli*, *K. pneumoniae*, and *P. aeruginosa* were the dominant species causing MDRO infections in ICU patients; among Gram-positive bacteria, *S. aureus* and *S. pneumoniae* were major pathogens. The APACHE II score at admission; comorbidities involving the cardiovascular, cerebrovascular, and pulmonary systems; a duration of mechanical ventilation >7 days; and the number of invasive procedures were independent risk factors for poor prognosis in ICU patients with MDRO infections.

The study revealed that MDRO strains were primarily isolated from sputum cultures, with respiratory infections being the most common site of infection, further emphasizing the respiratory system as a high-risk area for MDRO infections. This contrasts slightly with a study reporting the following distribution of infection sites in MDRO-infected individuals: 55% in the urinary tract, 35% in the lungs, and the remaining 10% in other sites (including the abdomen, skin, and soft tissues) (Magira et al., 2018). This discrepancy may stem from differences in patient populations, the intensity of invasive procedures such as respiratory support, infection prevention and control practices, or specimen collection strategies across different ICUs. In this study, patients commonly received respiratory interventions such as mechanical ventilation, increasing the risk of pulmonary infections. This clinical context aligns with the predominance of respiratory infections in the results and highlights the diagnostic value of sputum specimens in MDRO infection surveillance.

The results also showed a diverse array of pathogens causing MDRO infections in ICU patients. Gram-negative bacteria, particularly *E. coli*, *K. pneumoniae*, and *P. aeruginosa*, emerged as the predominant species. This finding is in alignment with previous research, highlighting the predominance of Gram-negative bacteria in MDRO infections (Fanelli et al., 2024). As reported by Wei et al., *P. aeruginosa* and *K. pneumoniae* are the most commonly isolated MDRO strains (Jiang et al., 2022); Rumbidzai et al. also noted *E. coli* and *K. pneumoniae* as frequently isolated bacteria (El Zein et al., 2025; Majangara et al., 2018). Although Gram-positive bacteria were less frequent, they still constituted a significant portion of infections, with *S. aureus* and *S. pneumoniae* being among the key pathogens. The clinical importance of these pathogens stems primarily from the treatment challenges posed by their resistance profiles. Both *S. aureus* and *S. pneumoniae* are Gram-positive facultative anaerobes capable of causing severe diseases, including bacteremia, pneumonia, and skin and soft tissue infections (Tamkin et al., 2025). Although environmental persistence [e.g., the long-term survival of *S. aureus* on surfaces (Gu et al., 2023)] may facilitate transmission, resistance is the core factor determining their clinical burden and prognosis. This study observed high resistance rates to commonly used antimicrobials among some Gram-positive strains (see Table 4), further underscoring the critical importance of implementing robust infection control measures (e.g., environmental disinfection to block transmission) and antimicrobial stewardship practices (to address resistance) in the ICU. Importantly, the study reveals significant variations in resistance rates among different pathogens, providing a crucial basis for clinical antibiotic selection. Physicians should prescribe antibiotics rationally based on the resistance characteristics of pathogens to improve treatment outcomes and reduce the emergence and spread of resistant organisms.

Through prognostic factor analysis, in this study, we identified six independent risk factors influencing 28-day survival: length of ICU stay, APACHE II score at admission, comorbid cardiovascular and cerebrovascular diseases, comorbid pulmonary diseases, mechanical ventilation duration >7 days, and the number of invasive procedures. The predictive model based on these factors demonstrated high discriminatory power (AUC = 0.913) and predictive accuracy (86.54%), with a sensitivity of 65.67% and specificity of 93.78%, offering significant guidance for early identification of high-risk patients in clinical practice. Kamuran et al. reported that longer hospital and ICU stays, prolonged mechanical ventilation, antimicrobial exposure, colonization status, invasive procedures, severity of underlying diseases, and reintubation are known factors increasing the risk of multidrug-resistant *A. baumannii* infections (Uluc et al., 2024). Clinically, the ICU is a high-risk area for MDRO colonization and infection (Li et al., 2024). Prolonged exposure to a resistant organism environment may accumulate the risk of MDRO infection over time. Additionally, a high APACHE II score indicates severe underlying diseases or multiorgan failure, leading to immunosuppression (Wang et al., 2024) and an inability to effectively clear invading MDROs. Jing et al. confirmed that sepsis patients with higher APACHE II scores at admission have increased 28-day mortality, which is associated with immune dysfunction (Wang et al., 2024). Comorbid cardiovascular, cerebrovascular, and pulmonary diseases increase susceptibility to MDRO infections and the risk of poor outcomes, likely due to chronic disease-induced persistent immunosuppression. For example, patients with heart failure and chronic lung diseases often exhibit systemic low-grade inflammation and T-cell exhaustion, impairing pathogen clearance (Halade and Lee, 2022; Lee et al., 2021). Additionally, swallowing and sputum excretion dysfunction in patients with cerebrovascular diseases (Nakamura et al., 2019) increases the risk of aspiration and colonization. Patients with cardiovascular diseases often require invasive procedures such as central venous catheters and arterial catheters, which disrupt skin and mucosal barriers and provide entry points for MDROs. Tracheal intubation (an invasive procedure) and mechanical ventilation have been proven to be independent risk factors for hospital-acquired infections in the ICU, with mechanical ventilation duration exceeding 7 days being a significant risk factor for ventilator-associated pneumonia caused by multidrug-resistant organisms (Jacob et al., 2020). Previous studies identified mechanical ventilation and urinary catheterization as risk factors for infections caused by MDROs (Han et al., 2022). Invasive procedures (e.g., catheterization, endoscopy, and surgery) require frequent contact with patient bodily fluids, and failure to strictly adhere to aseptic principles can easily lead to MDRO contamination of environmental surfaces. Furthermore, prolonged mechanical ventilation promotes biofilm formation on tracheal tube surfaces, providing a protective growth environment for MDROs. These patients often require broad-spectrum antibiotic therapy, leading to dysbiosis and an increased risk of MDRO colonization.

This study has several limitations that should be noted. First, the sample size was limited, and the data were derived from a single ICU, which may introduce selection and information bias, limiting the generalizability of the results. Second, we did not analyze the impact of antibiotic types, durations, and combination therapies on outcomes, potentially overlooking drug selection pressure as a key confounding factor. Additionally, the lack of whole-genome sequencing to analyze strain typing and resistance genes limits our understanding of MDRO transmission dynamics and outbreak potential.

## Conclusion

In summary, in our study, we show that respiratory infections are predominant among MDRO infections in ICU patients; Gram-negative bacteria, particularly *E. coli*, are the main pathogens, whereas Gram-positive bacteria include *S. aureus*; resistance rates vary significantly, necessitating individualized antimicrobial therapy. Prognostic analysis identified length of ICU stay; APACHE II score at admission; comorbid cardiovascular, cerebrovascular, and pulmonary diseases; mechanical ventilation duration; and the number of invasive procedures as independent prognostic predictors. The model demonstrated high discriminatory power and good predictive accuracy, aiding in the early identification of high-risk patients. These findings hold significant importance. The clarification of infection site distribution and pathogen spectra for MDROs in ICU patients provides a basis for targeted prevention and control. Additionally, variations in pathogen resistance suggest avoiding empirical use of broad-spectrum antibiotics. The identification of independent prognostic factors helps clinicians recognize high-risk patients at an early stage and implement intensive interventions to improve outcomes. The model’s high AUC value (0.913) indicates its potential to complement traditional scoring systems and support ICU resource allocation decisions. Given the limitations of this study, future research should conduct prospective multicenter studies to validate the external validity of our model, integrate molecular diagnostic techniques to analyze MDRO resistance genes and virulence factors, reveal transmission pathways and pathogenic mechanisms, and analyze antimicrobial prescription patterns combined with machine learning algorithms to predict resistance strain evolution trends and guide dynamic adjustments to antimicrobial stewardship programs.

## Data Availability

The data analyzed in this study are subject to the following licenses/restrictions: the experimental data used to support the findings of this study are available from the corresponding author upon request. Requests to access these datasets should be directed to Xiruiping5636@163.com.
